# Supplementary material for: Ancient hybridization leads to the repeated evolution of red flowers across a monkeyflower radiation
Source: Evol Lett. 2023 Jun 5;7(5):293–304. doi: 10.1093/evlett/qrad024 (PMC10565894; doi:10.1093/evlett/qrad024)
Supplement: qrad024_suppl_Supplementary_Material [file qrad024_suppl_supplementary_material.pdf]

Table S1. Sample information for the 47 sequenced individuals used in this study. The table includes taxon identity, sampling location, percent read alignment, and average sequencing depth. Samples in red text were sequenced as a part of this study, while those in black were described in Stankowski et al (2019).

Sample	Taxon	Latitude	Longitude	% Reads aligned	Seq. Depth
**159_83**	ssp. *aridus*	32.6630	-116.2230	91.7	21.12
**159_84**	ssp. *aridus*	32.6630	-116.2230	89.3	21.98
**195_1**	ssp. *aridus*	32.6300	-116.1429	92.6	20.20
**T84**	ssp. *aridus*	32.6526	-116.2449	87.2	21.75
**T102**	ssp. *aurantiacus*	39.0424	-122.7727	94.9	23.74
**T104**	ssp. *aurantiacus*	39.2045	-123.7646	94.6	25.09
**T50**	ssp. *aurantiacus*	35.9865	-121.4928	88.3	24.36
**T92**	ssp. *aurantiacus*	37.8459	-120.6110	94.0	15.16
**T144**	ssp. *calycinus*	34.1929	-117.2784	93.2	26.00
**T150**	ssp. *calycinus*	33.8564	-116.8481	94.7	24.02
**T90**	ssp. *calycinus*	35.5918	-118.5052	91.3	19.97
**T91**	ssp. *calycinus*	35.3172	-118.5871	95.5	27.91
**T101**	ssp. *grandiflorus*	39.5536	-121.4301	92.0	16.05
**T61**	ssp. *grandiflorus*	39.5590	-120.8243	91.6	17.31
**T96**	ssp. *grandiflorus*	39.0122	-120.7552	92.0	28.21
**T99**	ssp. *grandiflorus*	39.4376	-121.0599	91.4	23.84
**DPR_Y3**	ssp. *longiflorus*, yellow	33.7459	-117.4485	96.0	26.88
**SS_Y16**	ssp. *longiflorus*, yellow	34.2722	-118.6100	94.2	30.86
**T33_1**	ssp. *longiflorus*, yellow	34.3438	-118.5099	94.6	18.87
**T8_8**	ssp. *longiflorus*, yellow	34.1347	-118.6452	82.6	25.11
**DPR_R12**	ssp. *longiflorus*, red	33.7459	-117.4485	89.8	18.52
**MBR_R3**	ssp. *longiflorus*, red	34.2056	-117.6765	94.4	23.20
**SS_R19**	ssp. *longiflorus*, red	34.2722	-118.6100	91.3	23.89
**WTF_R1**	ssp. *longiflorus*, red	34.2174	-117.7630	94.2	23.45
**KK168**	ssp. *parviflorus*	34.0180	-119.6730	91.8	23.66
**KK161**	ssp. *parviflorus*	34.0180	-119.6730	92.0	19.11
**KK180**	ssp. *parviflorus*	34.0180	-119.6730	92.4	18.18
**KK182**	ssp. *parviflorus*	34.0193	-119.6802	91.3	19.46
**MTH17**	ssp. *puniceus*, *OC*, red	33.6414	-117.8252	90.6	21.23
**MTH15**	ssp. *puniceus*, *OC*, red	33.6414	-117.8252	91.1	18.85
**RPR16**	ssp. *puniceus*, *OC*, red	33.6058	-117.8020	95.1	21.68
**LFP13**	ssp. *puniceus*, *OC*, yellow	33.6532	-117.6579	90.3	22.23
**VCR14**	ssp. *puniceus*, *OC*, yellow	33.4871	-117.6489	92.2	19.97
**VCR17**	ssp. *puniceus*, *OC*, yellow	33.4871	-117.6489	90.7	21.38
**ELF**	ssp. *puniceus*, red	33.0860	-117.1453	93.0	18.20

**JMC**	ssp. *puniceus*, red	32.7373	-116.9541	93.8	19.06
**LH**	ssp. *puniceus*, red	33.0609	-117.1188	87.1	19.77
**MT**	ssp. *puniceus*, red	32.8210	-117.0618	93.7	20.85
**UCSD**	ssp. *puniceus*, red	32.8894	-117.2362	87.0	18.23
**BCRD**	ssp. *puniceus*, yellow	32.9496	-116.6380	94.6	20.85
**INJ**	ssp. *puniceus*, yellow	33.0979	-116.6643	93.1	18.83
**LO**	ssp. *puniceus*, yellow	32.6767	-116.3312	93.4	18.04
**PCT**	ssp. *puniceus*, yellow	32.7326	-116.4698	92.3	19.68
**POTR**	ssp. *puniceus*, yellow	32.6038	-116.6339	90.5	19.27
**CLV_GH**	*M. clevelandii*	33.1589	-116.8122	92.3	21.31
**CLV_11**	*M. clevelandii*	33.3391	-116.9325	84.4	15.52
**CLV_4**	*M. clevelandii*	33.3391	-116.9325	89.3	17.31

Table S2. Patterson's D statistics, estimated using the *Dtrios* function in the *Dsuite* package, for all trios of taxa included in this study. In all cases, *M. clevelandii* is used as the outgroup, and the three ingroup taxa are indicated (P1, P2, P3). The identity of the P1 and P2 taxon is adjusted, so that the D-statistics are always positive. Counts of each site category (BBAA, ABBA, and BABA) are indicated.

P1	P2	P3	Dstatistic	Z-score	p-value	f4-ratio	BBAA	ABBA	BABA
aurantiacus	*calycinus*	*aridus*	0.0247	3.5809	3.4236E-04	0.0085	503634	144599	137624
longiflorus	*calycinus*	*aridus*	0.0231	5.6748	1.3888E-08	0.0066	585489	120190	114762
aurantiacus	*longiflorus*	*aridus*	0.0055	0.7555	4.4995E-01	0.0019	500695	140659	139113
aurantiacus	*OC*	*aridus*	0.0393	3.4859	4.9054E-04	0.0140	484366	152419	140898
calycinus	*OC*	*aridus*	0.0169	2.0655	3.8877E-02	0.0056	531413	136476	131930
longiflorus	*OC*	*aridus*	0.0389	4.2424	2.2114E-05	0.0122	545247	133213	123239
aurantiacus	*Red ecotype*	*aridus*	0.0720	5.5712	2.5299E-08	0.0264	476747	161375	139700
calycinus	*Red ecotype*	*aridus*	0.0524	4.8891	1.0132E-06	0.0180	515736	147655	132955
longiflorus	*Red ecotype*	*aridus*	0.0743	6.3053	2.8760E-10	0.0245	525314	145558	125430
OC	*Red ecotype*	*aridus*	0.0440	6.5784	4.7556E-11	0.0125	590243	120491	110336
aurantiacus	*Yellow ecotype*	*aridus*	0.0976	7.9287	2.2138E-15	0.0366	472730	169291	139178
calycinus	*Yellow ecotype*	*aridus*	0.0817	7.9242	2.2968E-15	0.0284	517873	153126	129988
longiflorus	*Yellow ecotype*	*aridus*	0.1033	9.5022	0.0000E+00	0.0348	524357	152595	124029
OC	*Yellow ecotype*	*aridus*	0.0741	11.6458	0.0000E+00	0.0229	563239	134736	116144
Red ecotype	*Yellow ecotype*	*aridus*	0.0354	5.3659	8.0570E-08	0.0105	586759	123454	115016
longiflorus	*calycinus*	*aurantiacus*	0.0142	3.0375	2.3856E-03	0.0152	264814	158551	154123
OC	*calycinus*	*aurantiacus*	0.0646	12.9041	0.0000E+00	0.0728	226218	185771	163228
Red ecotype	*calycinus*	*aurantiacus*	0.0810	13.8921	0.0000E+00	0.0916	217043	193298	164335
Yellow ecotype	*calycinus*	*aurantiacus*	0.0914	16.1788	0.0000E+00	0.1016	222638	193789	161330
OC	*longiflorus*	*aurantiacus*	0.0542	7.6557	1.9227E-14	0.0585	238017	176044	157929
Red ecotype	*longiflorus*	*aurantiacus*	0.0710	9.0906	1.0842E-19	0.0776	224813	184963	160429
Yellow ecotype	*longiflorus*	*aurantiacus*	0.0810	12.6167	0.0000E+00	0.0878	227424	187130	159100
aridus	*parviflorus*	*aurantiacus*	0.1961	14.0213	0.0000E+00	0.1348	285314	268360	180373
Red ecotype	*OC*	*aurantiacus*	0.0230	4.3617	1.2905E-05	0.0203	290560	142601	136181
Yellow ecotype	*OC*	*aurantiacus*	0.0330	4.9649	6.8722E-07	0.0310	270354	155205	145289
Yellow ecotype	*Red ecotype*	*aurantiacus*	0.0126	2.2007	2.7757E-02	0.0109	297206	140834	137339
aridus	*parviflorus*	*calycinus*	0.1748	10.9417	0.0000E+00	0.1579	285807	267415	187841
Red ecotype	*OC*	*calycinus*	0.0555	9.6966	0.0000E+00	0.1384	243727	158758	142055
Yellow ecotype	*OC*	*calycinus*	0.0356	6.1916	5.9553E-10	0.1002	220904	168746	157148
Red ecotype	*Yellow ecotype*	*calycinus*	0.0171	3.8015	1.4382E-04	0.0421	250570	152011	146907
aridus	*parviflorus*	*longiflorus*	0.1850	12.9018	0.0000E+00	0.1572	287491	267691	184097
Red ecotype	*OC*	*longiflorus*	0.0723	14.5433	0.0000E+00	0.1678	231928	164056	141932
Yellow ecotype	*OC*	*longiflorus*	0.0656	13.0470	0.0000E+00	0.1650	211269	176209	154529
Red ecotype	*Yellow ecotype*	*longiflorus*	0.0015	0.3270	7.4369E-01	0.0034	242556	151013	150569
calycinus	*aurantiacus*	*parviflorus*	0.0048	0.6611	5.0857E-01	0.0024	428154	150130	148692
longiflorus	*aurantiacus*	*parviflorus*	0.0095	1.0433	2.9681E-01	0.0047	425178	151583	148737
OC	*aurantiacus*	*parviflorus*	0.0213	2.2775	2.2757E-02	0.0107	412604	157123	150556
Red ecotype	*aurantiacus*	*parviflorus*	0.0214	2.5212	1.1695E-02	0.0108	406985	157924	151306
Yellow ecotype	*aurantiacus*	*parviflorus*	0.0199	2.3536	1.8592E-02	0.0102	405744	160179	153933
longiflorus	*calycinus*	*parviflorus*	0.0057	0.9576	3.3825E-01	0.0023	509818	124093	122685
OC	*calycinus*	*parviflorus*	0.0186	2.9785	2.8963E-03	0.0084	460163	140254	135125
Red ecotype	*calycinus*	*parviflorus*	0.0183	3.2225	1.2706E-03	0.0084	447078	143871	138691
Yellow ecotype	*calycinus*	*parviflorus*	0.0170	3.2455	1.1726E-03	0.0078	451949	143638	138830
OC	*longiflorus*	*parviflorus*	0.0139	1.8662	6.2017E-02	0.0061	473694	135280	131558
Red ecotype	*longiflorus*	*parviflorus*	0.0137	2.5911	9.5673E-03	0.0061	456355	140065	136292
Yellow ecotype	*longiflorus*	*parviflorus*	0.0122	2.2038	2.7536E-02	0.0055	457988	141254	137854
Red ecotype	*OC*	*parviflorus*	0.0002	0.0429	9.6576E-01	0.0001	524946	114939	114888
OC	*Yellow ecotype*	*parviflorus*	0.0013	0.2585	7.9602E-01	0.0005	500637	123762	123441
Red ecotype	*Yellow ecotype*	*parviflorus*	0.0016	0.6136	5.3950E-01	0.0006	527218	115540	115168
calycinus	*longiflorus*	*OC*	0.0521	6.8952	5.3776E-12	0.0921	221249	172531	155434
aridus	*parviflorus*	*OC*	0.1534	8.9739	3.2526E-19	0.1204	286312	262790	192892
Yellow ecotype	*Red ecotype*	*OC*	0.1006	13.8492	0.0000E+00	0.2457	181411	179420	146609
calycinus	*longiflorus*	*Red ecotype*	0.0368	4.7774	1.7761E-06	0.0725	235255	164413	152737
aridus	*parviflorus*	*Red ecotype*	0.1300	7.2320	4.7589E-13	0.1129	283071	259498	199805
calycinus	*longiflorus*	*Yellow ecotype*	0.0220	3.1743	1.5023E-03	0.0576	233650	163251	156236
aridus	*parviflorus*	*Yellow ecotype*	0.1114	6.1979	5.7217E-10	0.1062	280770	257570	205942

Table S3. The *t*-ratio from a linear mixed-effects model showing the difference in admixture proportion (f_d) using different taxa as P1, with the yellow ecotype as P2, *aridus* as P3 and *M. clevelandii* as the outgroup. Statistical significance is denoted as: *** for p < 0.001, ** for p < 0.01 and * for p < 0.05.

**Factor Level**	**Estimate**	***df***	***t*-ratio**
aurantiacus vs *calycinus*	0.016113	11102	6.945***
aurantiacus vs *longiflorus*	0.015227	11102	6.65***
aurantiacus vs *OC*	0.025113	11102	10.757***
aurantiacus vs red ecotype	0.036214	11102	15.363***
calycinus vs *longiflorus*	-0.000885	11102	-0.387
calycinus vs *OC*	0.009	11102	3.862**
calycinus vs red ecotype	0.020101	11102	8.542***
longiflorus vs *OC*	0.009885	11102	4.298***
longiflorus vs *OC*	0.020987	11102	9.033***
OC vs red ecotype	0.011102	11102	4.69***

Table S4. The *t*-ratio from a linear mixed-effects model showing the difference in admixture proportion (f_d) due to recombination rate. Quantile bins of recombination rate (in cM/Mb) are presented. Statistical significance is denoted as: *** for p < 0.001, ** for p < 0.01, and * for p < 0.05.

**Factor Level**	**Estimate**	***df***	***t*-ratio**
[0,0.824] vs (0.824,1.51]	-0.001286	10577	-0.526
[0,0.824] vs (1.51,2.33]	-0.001071	10577	-0.446
[0,0.824] vs (2.33,3.66]	-0.020672	10577	-8.687***
[0,0.824] vs (3.66,10.9]	-0.035771	10577	-15.431***
(0.824,1.51] vs (1.51,2.33]	0.000215	10577	0.089
(0.824,1.51] vs (2.33,3.66]	-0.019386	10577	-8.089***
(0.824,1.51] vs (3.66,10.9]	-0.034486	10577	-14.766***
(1.51,2.33] vs (2.33,3.66]	-0.019601	10577	-8.33***
(1.51,2.33] vs (3.66,10.9]	-0.034700	10577	-15.149***
(2.33,3.66] vs (3.66,10.9]	-0.015099	10577	-6.654***

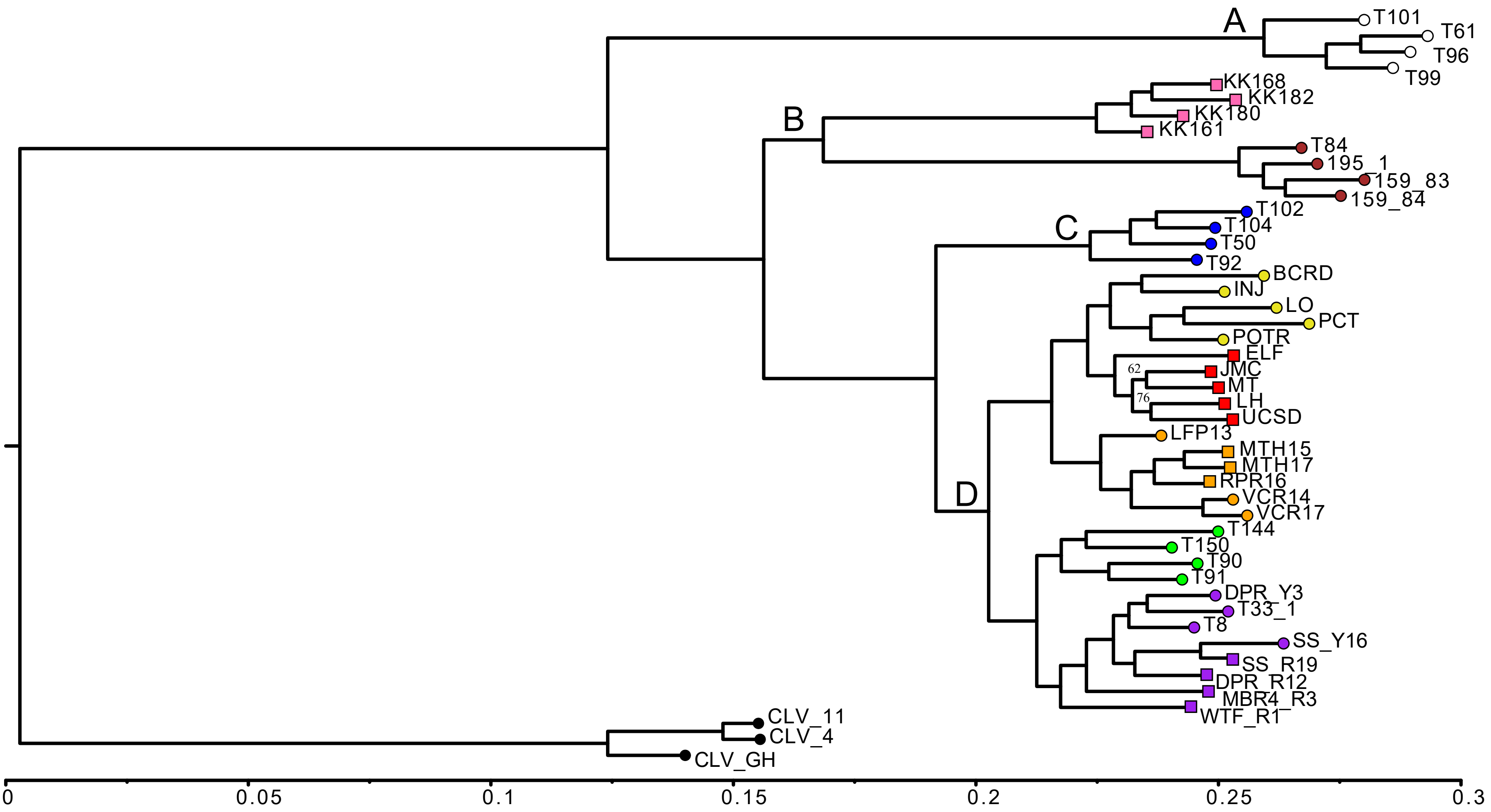

Fig. S1. Maximum likelihood consensus species tree generated from all variant sites among the 47 samples and estimated using IQ-TREE v1.6.12. Colors at the tips vary by taxon, and are the same as used in Fig 1 of the main text. Circles correspond to plants with yellow flowers and squares denote plants with red flowers. The four primary clades are noted with letters A-D. Nodes show 100% support from 1000 bootstrap replicates, except where indicated.

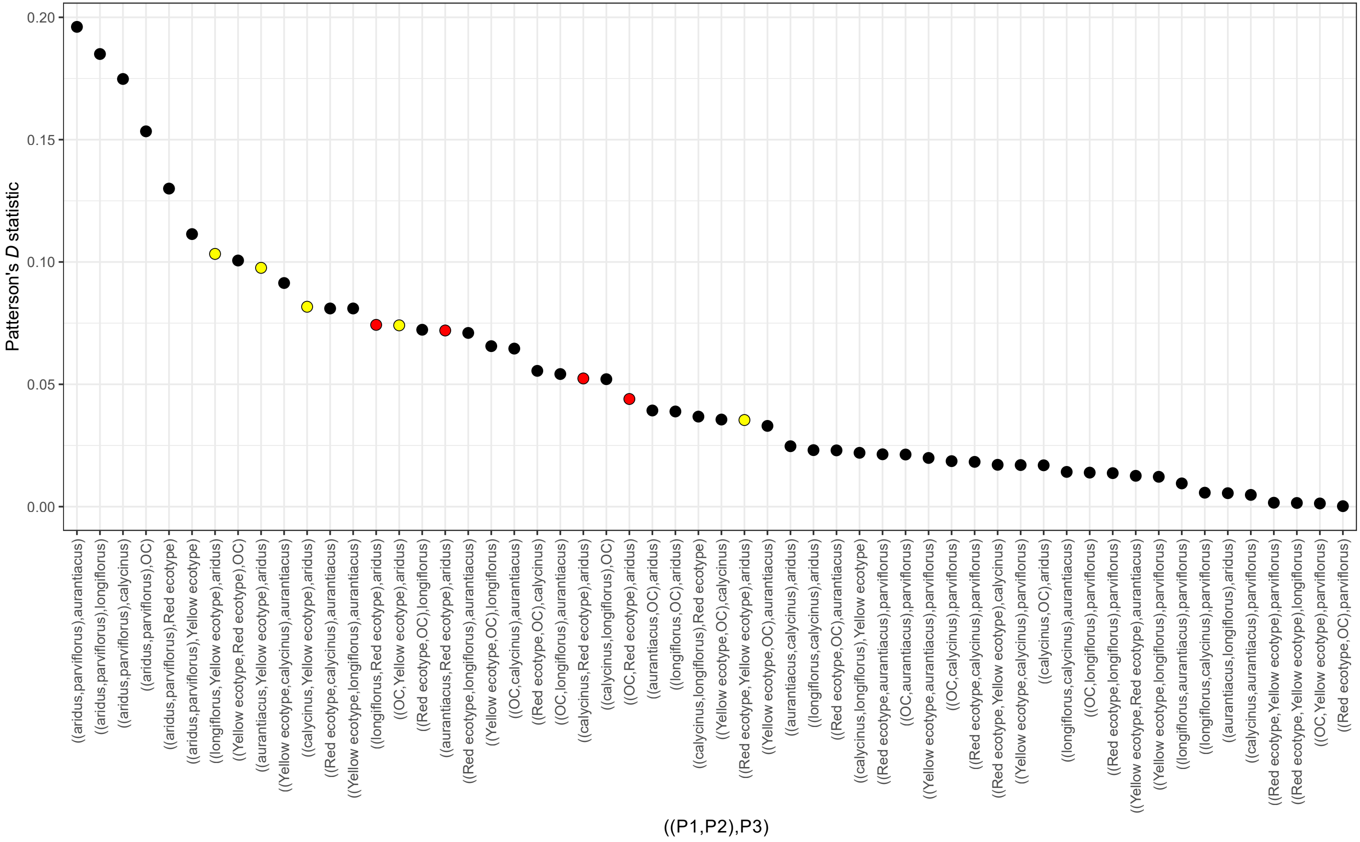

Fig. S2. The distribution of Patterson's D statistic among all calculated trios. The tests that are included in Table 1 are colored red or yellow, depending on whether the red ecotype or yellow ecotype was set as P2.

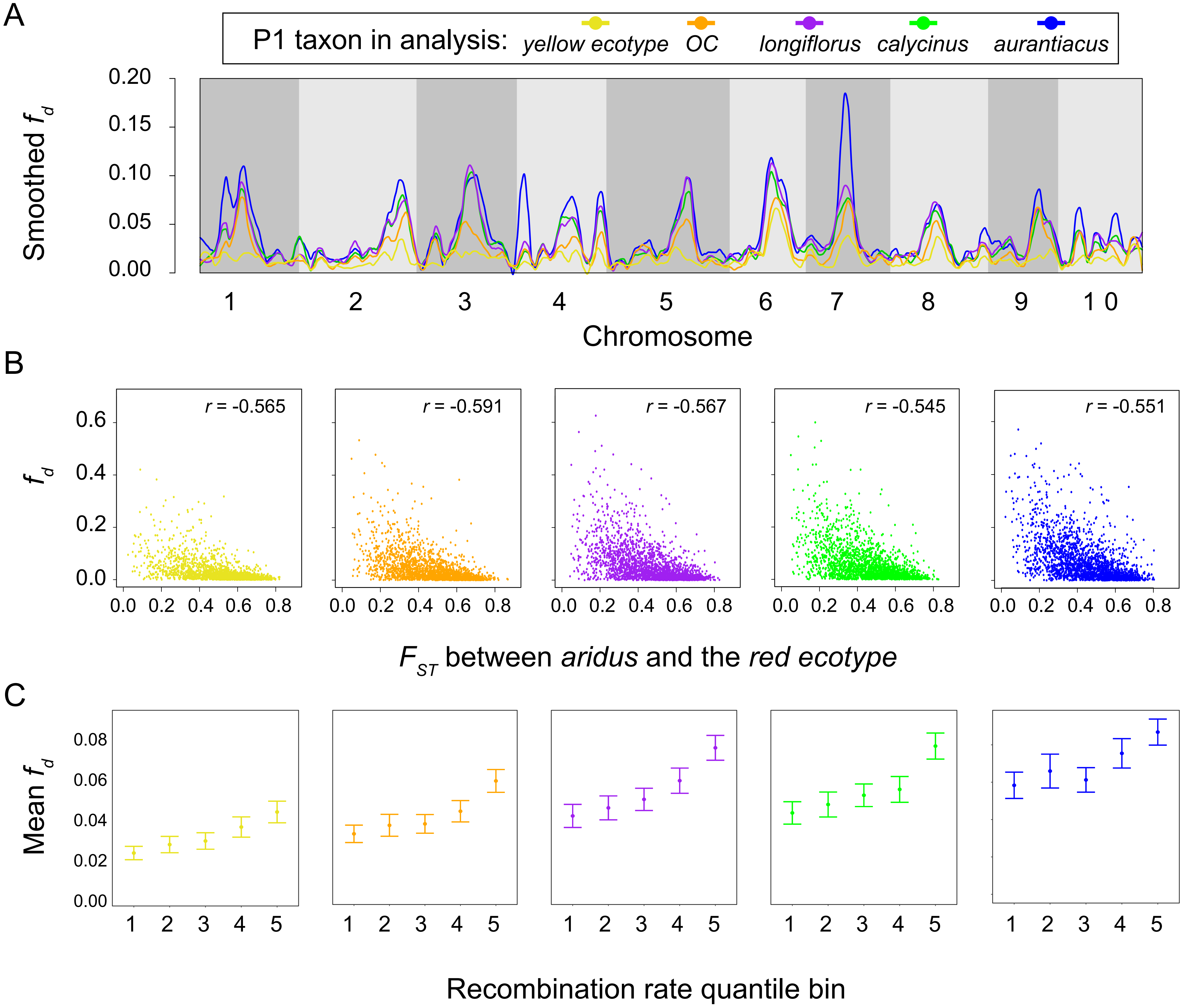

Figure S3. (A) Loess-smoothed fd values plotted across the 10 chromosomes of the Mimulus aurantiacus genome, with the red ecotype as P2, aridus as P3, and one of five different taxa as P1. Colors indicate the taxa from clades C and D used as P1. (B) Scatterplots showing the relationship between Fst and fd in these same 50 kb windows, with the correlation coefficient between the statistics in the upper right-hand corner of each plot. (C) Mean and 95% confidence intervals of fd in different quantile bins of recombination rate. Quantile bins of recombination rate are as follows: 1 = 0 - 0.824 cM/Mb; 2 = 0.824 - 1.51 cM/Mb; 3 = 1.51 - 2.33 cM/Mb; 4 = 2.33 - 3.66 cM/Mb; 5 = 3.66 - 10.9 cM/Mb.

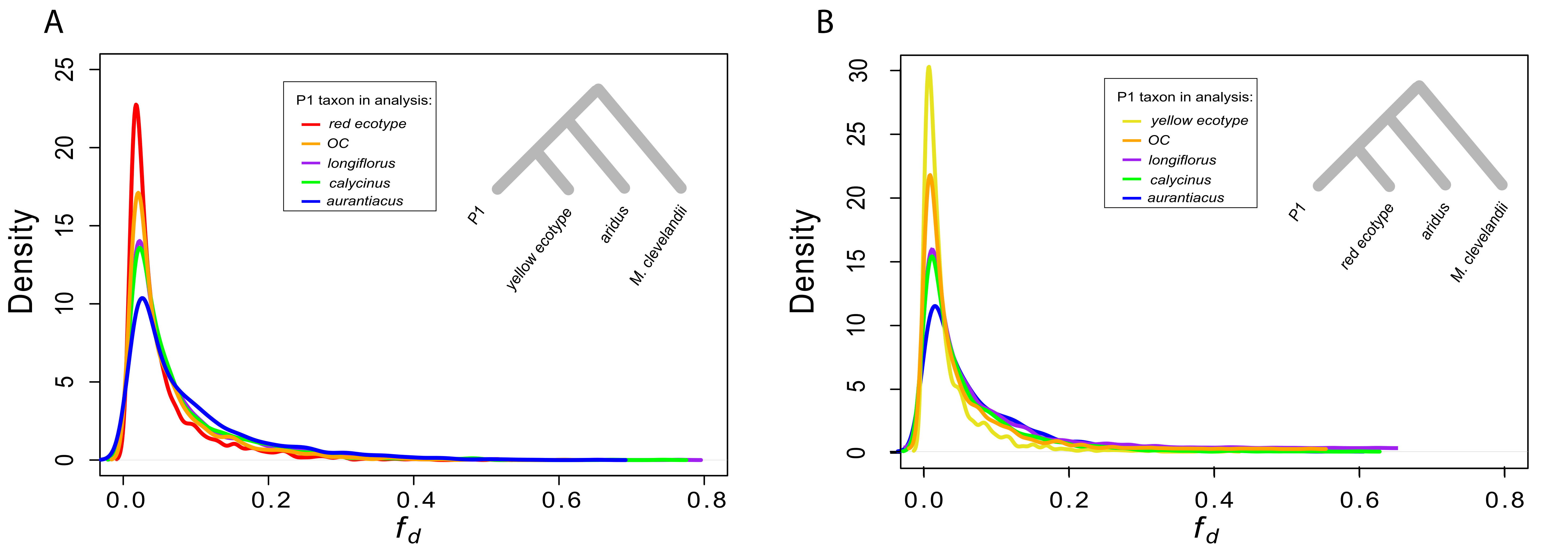

Fig. S4. Density plots of f_d, calculated in 50 kb windows across the genome. A) The P1 taxon varied in each test, with P2 set as the yellow ecotype, P3 set as *aridus*, and the outgroup is *M. clevelandii*. B) The P1 taxon varied in each test, with P2 set as the red ecotype, P3 as *aridus*, and the outgroup is *M. clevelandii*.

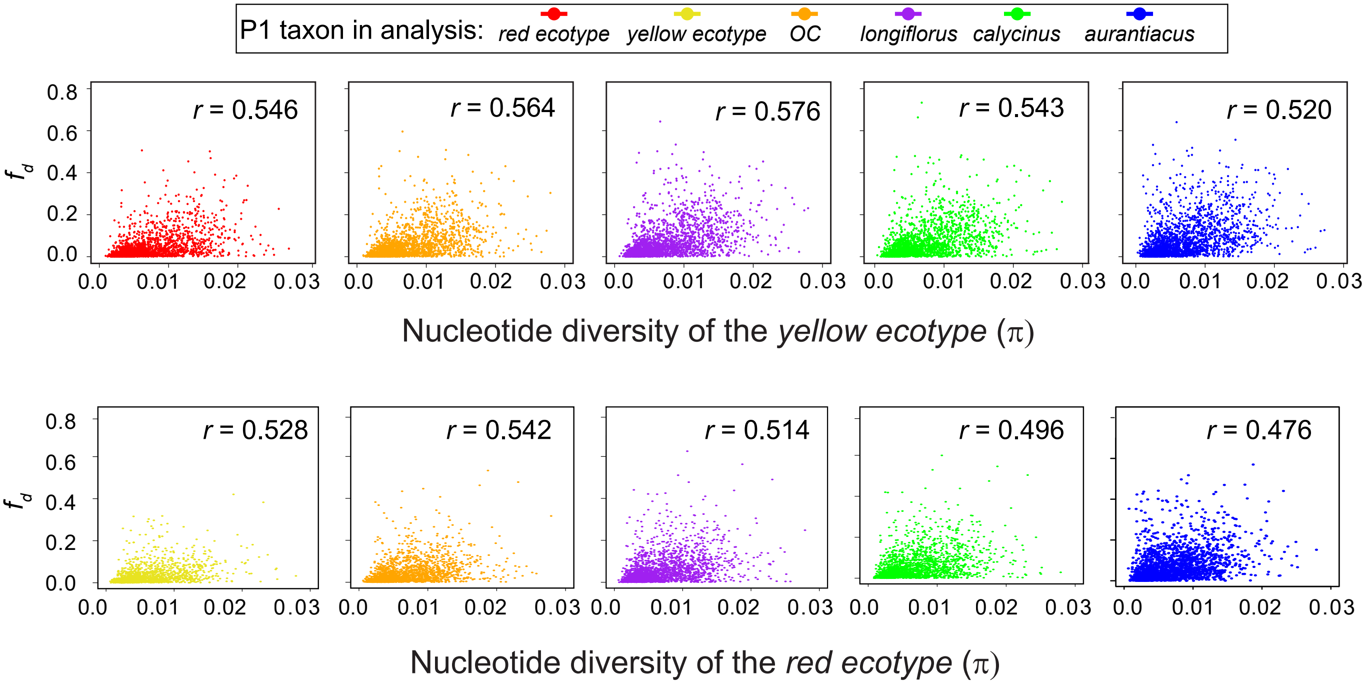

Fig. S5. Scatterplots and correlations between f_d and π in 50 kb windows. Top, f_d is calculated with different P1 taxa, the yellow ecotype as P2, *aridus* as P3, and *M. clevelandii* as the outgroup. Bottom, f_d is calculated with different P1 taxa, the red ecotype as P2, *aridus* as P3, and *M. clevelandii* as the outgroup.

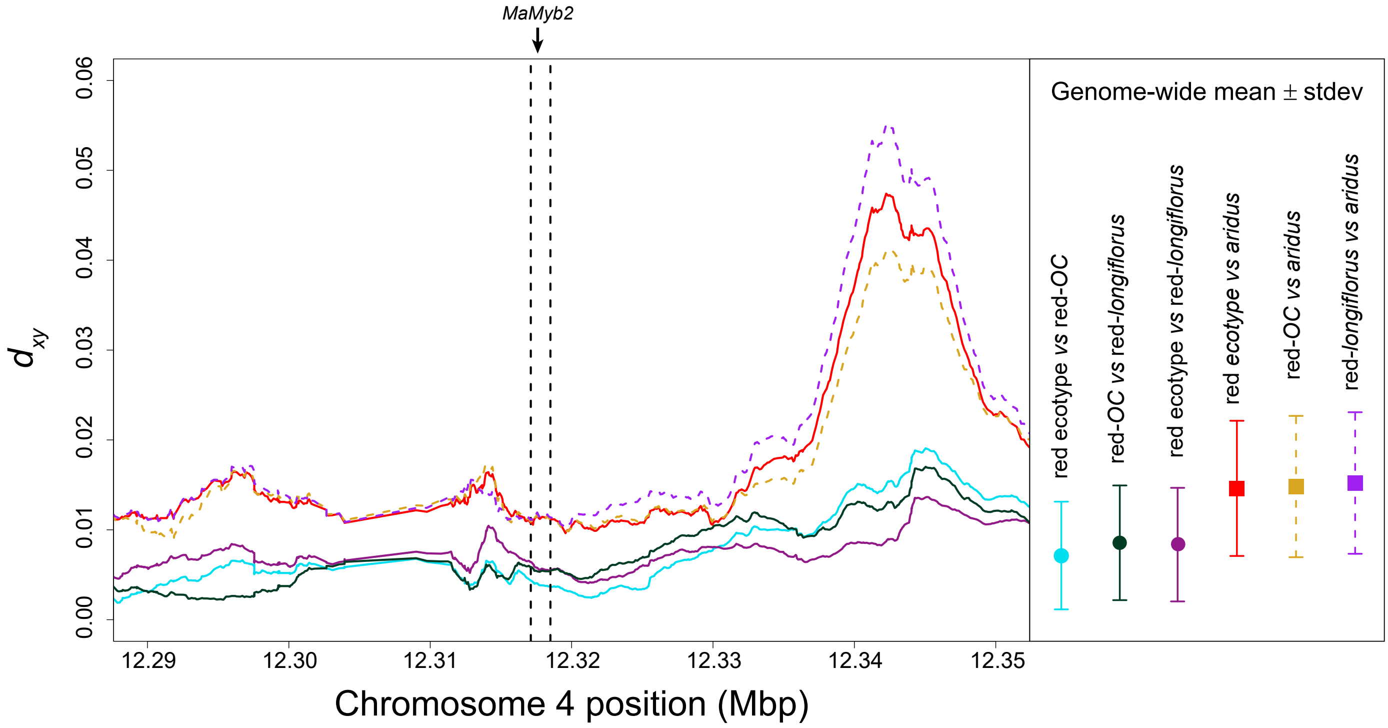

Fig. S6. Scans of d_{xy} along chromosome 4, in the vicinity of *MaMyb2*. Divergence is calculated in overlapping 10 kb windows (100 bp steps) either between each of the three red-flowered taxa and *aridus*, or between each of the red-flowered taxa. The genome-wide mean values of d_{xy} (error bars = standard deviations) are presented to the right, with the color scheme used the same as the scans.
